# Bumblebee hairs as electric and air motion sensors: theoretical analysis of an isolated hair

**DOI:** 10.1098/rsif.2020.0146

**Published:** 2020-07-08

**Authors:** K. Koh, D. Robert

**Affiliations:** School of Biological Sciences, University of Bristol, Bristol, UK

**Keywords:** mechanoreceptor, electroreceptor, air flow, electrostatics

## Abstract

Foraging bumblebees are electrically charged. Charge accumulation has been proposed to enable their ability to detect and react to electrical cues. One mechanism suggested for bumblebee electro-sensing is the interaction between external electric fields and electric charges accumulating on fine hairs on the cuticular body. Such hairs exhibit several functional adaptations, for example, thermal insulation, pollen capture and notably, the sensing of air motion such as flow currents or low frequency sound particle velocity. Both air motion and electric fields are ubiquitous in the sensory ecology of terrestrial arthropods, raising the question as to whether cuticular hairs respond to both stimuli. Here, a model-theoretical approach is taken to investigate the capacity of bumblebee filiform hairs as electric sensors and compare it to their response to air motion. We find that oscillating air motion and electric fields generate different mechanical responses, depending on stimulus frequency and body geometry. Further, hair morphology can enhance one sensing mode over the other; specifically, higher surface area favours electric sensitivity. Assuming a maximum stable charge on the hair that is limited only by electric breakdown of air, it is expected that an applied oscillating electric field strength of approximately 300 V m^−1^ produces comparable mechanical response on the hair as a 35 mm s^−1^ air flow oscillating at 130 Hz—an air disturbance signal similar to that produced by wingbeats of insects within a few bodylengths of the bumblebee. This analysis reveals that bumblebee filiform hairs can operate as bi-modal sensors, responding to both oscillating electric and air motion stimuli in the context of ecologically relevant scenarios.

## 1. Introduction

Bees typically possess a net positive electric charge [1–3]. The electric field associated with this charge exerts an electrostatic force on nearby charged or polarizable materials, for example, pollen. This electrostatic force has been proposed to enhance pollen collection and efficiency of pollination [1,4–6]. Experimental studies have shown that bumblebee foraging behaviour can be altered by variations in electric fields of similar amplitude to those surrounding flowers, evidencing electric sensing in bumblebees [3]. This electric sensing ability has also been proposed to be affected by the electric charge carried by the bee. For honeybees and bumblebees respectively, the putative organs of electroreception are proposed to be the antennae and hairs, functioning as electro-mechanical transducers [7,8]. For both antennae and hairs, the presence of net charge was shown to increase the mechanical response generated by electric field stimuli.

A key characteristic of pollinators such as bees is that they present a variety of hairs with different dimensions, structures and functions. One of the main recognized functions is thermal insulation (e.g. [9,10]). Many bee hairs exhibit branching structures that help trap pollen grains [11]. In addition to mechanical trapping action, electrostatic forces have been invoked to facilitate the process of pollen collection by attracting charged pollen grains towards the bee. Interestingly, only some of the hairs borne on the insects’ cuticle are innervated by basal mechanoreceptive neurons [8,12].

Commonplace across arthropods, basally articulated filiform hairs undergo mechanical displacement in response to weak air motion from air flow currents and sound particle velocity [13–15]. Such motion can be quite small, sometimes nanoscale, and still trigger an electrophysiological response from the basal mechano-sensory neurons. Detection of air motion in nature encodes useful information from conspecifics, predators or meteorological conditions that confer adaptive value to the beholder [16]. In honeybees, acoustic air vibration signals are proposed to be a medium for communications in the hive and are picked up by the antennae [17]. In contrast, the ecological relevance of electrosensing in bees remains to be explored and tested in natural conditions. In bumblebees, electric and air motion sensing can be achieved using the same morphological substrate, i.e. filiform mechano-sensory hairs [8]. Questions then arise with respect to receptor design, in particular as to whether electric field and air motion command different response characteristics and stimulus specificity. Do filiform hairs exhibit different responses for each sensory modality, encoding specific electric and air motion information separately?

Previous work [8] establishes the capacity of electroreception via the hair mechano-sensor. Whether the bumblebee hairs considered are also sensitive to air motion, endowing them with dual function, has not been addressed. Here, the intention is to compare electrical to air motion effects on hair movement so as to provide a starting point for answering the questions posed above.

This work takes a theoretical approach, focusing on the bumblebee hair mechano-sensor, investigating in some detail the likely physical mechanism behind electro-mechanical coupling. The first objective is to identify the key parameters of hair electric field sensing and their theoretical limits. Secondly, a comparison is drawn between electric and air motion stimuli and their coupling to the hair receptor.

The hair mechano-sensor is first reduced to a simple model of an isolated hair (§2) which allows a first order approximation of torque generated from either electric field (§3) or air motion forces (§4). Only oscillating forces are considered, which means that for the mechano-sensory hair, the resultant angular displacement is proportional to the applied oscillating torque. This allows a comparison of the hair mechano-sensory response due to electric field and air motion stimuli by considering only the torque applied (§5). Air motion torque on the hair is estimated using realistic stimulus signal parametrized from the ecologically relevant scenario of air disturbance from nearby insect wingbeats. Taking this to be a perceivable stimulus, the electric field stimulus required to produce a similar hair response is calculated (§6). This theoretical approach offers a deeper understanding of hair-based electro-mechanical sensing and enables a fundamental generalizable modelling framework that will guide further research into electro-reception in air and its sensory ecological relevance.

## 2. Bee hair mechano-sensor model

Mechano-sensory hairs are often described mechanically as a pendulum, a rod pivoting around a hinge located where the hair joins the body [13,14,18–20]. This hinge consists of nerve cells that serve to convert mechanical motion to electrophysiological signals. The hair and hinge together form a mass–spring–damper system with characteristic frequency response. For an air motion sensor, the hinge stiffness is usually low compared the hair bending stiffness so that the hair moves as a stiff rod about the hinge [14,21].

**Figure 1.**
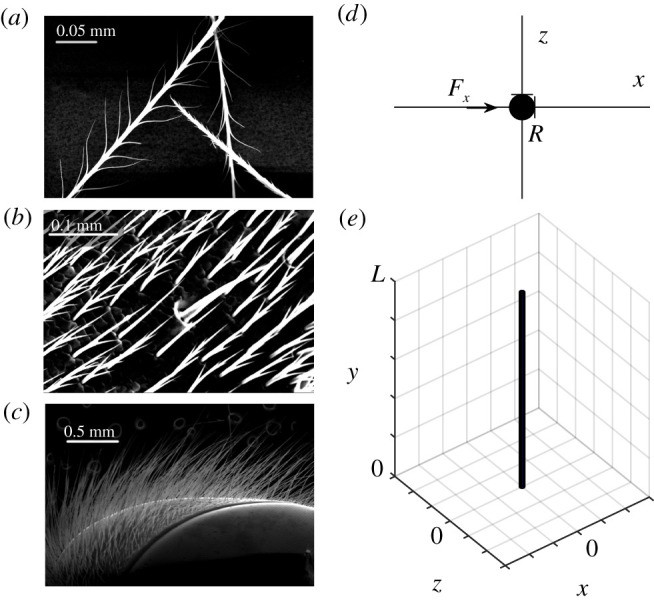
Bumblebee hair. (*a*–*c*) Scanning electron microscopy images of real bumblebee hair showing branching, tapering hairs arranged in tight arrays. (*d*) Cross-section of the model cylindrical hair in the *xz* plane; the direction of the force considered is shown (*F*_*x*_ along the *x* axis). (*e*) Illustration of the cylindrical hair and coordinate system.

To make calculating the force tractable, the mechano-sensory hair is approximated to a stiff, cylindrical rod basally attached to the body. The torque on this model hair generated from an applied force, **F**, can be calculated as, $T=\int_{0}^{L}F(l)\times l\;\text{d}l$, where **l** is the vector describing the line between the point of force application and the base of the hair at the origin. Figure 1 illustrates the model hair and coordinate system.

Some simplifications are made here:

1. Hair length is much greater than its diameter, *L* ≫ 2*R* (typical R=1−4 μm and L=0.1−1.5 mm), so effects from the ends of the cylinder are negligible.
2. Hair geometrical features such as branching and tapering are not considered, otherwise scanning electron micrographs of bumblebee hairs document that a cylindrical geometry is a reasonable approximation of filiform hair morphology (figure 1).
3. The hair does not bend along its length.
4. Hair displacement response about the hinge is small, of the same or smaller length scale as 2*R*, so that the effect on force coupling is negligible, an assumption that is considered valid for hair mechano-sensors [14].

With these simplifications and considering only the component of the force perpendicular to the hair *F*_*x*_ (figure 1), the vector notation can be dropped, subsequently,

2.1 $$T=\int_{0}^{L}F_{x}y\;\text{d}y.$$

This applies to a Cartesian coordinate system with axes *x*, *y* and *z*. The axis of the cylidrical hair lies along the *y* axis and only the perpendicular force along the *x* axis, *F*_*x*_, is considered.

## 3. Oscillating electric force

In the presence of an electric field *E*, the charges *q* on a bee hair will experience an electric (Coulomb) force *F*_*E*_ = *qE*. The torque generated by the electric force on a charged hair, *T*_*E*_, can be estimated simply by substituting *F*_*E*_ for *F*_*x*_ in equation (2.1). If some simplifying assumptions are made:

1. Charge on the cylindrical hair (§2) resides on the surface with uniform density, *σ* C m^−2^, so the total charge on the hair is 2*σπR L*.
2. Surface conductivity of the hair is low so that charges are fixed in position.
3. *E* is spatially homogeneous.

The integral for *T*_*E*_ can be solved:

3.1 $$T_{E}=\sigma \pi RL^{2}E_{x}.$$

Note that only an oscillating electric field perpendicular to the hair in the direction of the *x* axis is considered, *E*_*x*_ = |*E*_*x*_|sin *ωt*, where *ω* is angular frequency and *t* is time, so it can be compared to an oscillating air motion stimulus (see §§4 and 5).

### 3.1. Maximum electric torque

Equation (3.1) shows that the torque of the cylindrical hair mechano-sensor due to the electric field is directly proportional to the surface area of the hair and surface charge density *σ*. There has been no reported observation of *σ* on individual hairs. Thus far, measurements of charge have only been reported for whole bees. It would be wrong to assume homogeneous charging of the bee to estimate *σ* of a single hair. Hence, *T*_*E*_ on the hair cannot be estimated directly. Instead, physical constraints on *σ* can be explored to find limitations on possible |*T*_*E*_|.

Typically for a solid surface, *σ* is limited by the dielectric breakdown of air. If *σ* is small, the electric field of the charges is weak, in which case, discharge through air is Ohmic. Due to the low conductivity of air (approx. 10^−15^ S m^−1^), the discharge rate is typically low, so charge can accumulate. When charges accumulate to the point where their electric field exceeds the air dielectric breakdown threshold *E*_*b*_, electrons in the air are accelerated by the field to ionizing energies so that collisions with air molecules produce more electrons. This process continues with the electrons that are produced so that the number of electrons in the air increases exponentially by a factor

3.2 Q=exp∫α dl,

where *α* is the effective ionization coefficient that is dependent on the electric field strength and *l* is the path of the electric field. This is the main mechanism of air dielectric breakdown and the electron number density enhancement results in a significant increase in air conductivity [22,23] and *σ* cannot increase further.

For a spatially uniform static or low frequency electric field in ground level air, *E*_*b*_ ≈ 3 MV m^−1^ and the corresponding *σ* = 27 μC m^−2^. However, the electric field of charge on the surface of cylinders of small radius (less than 1 mm) can significantly exceed 3 MV m^−1^ without breakdown [23, fig. 3]. This is attributed to the non-uniform electric field of charges on a cylindrical surface which decreases with radial distance [24]:

3.3 $$E_{c}(x)=\frac{\sigma }{\varepsilon \varepsilon_{0}}\frac{R}{x},$$

where ɛ_0_ is the electrical permittivity of free space and ɛ is the relative permittivity of the material (air in this case). Equation (3.3) implies that for small *R* any strong electric field is only sustained in a thin sheath around the cylinder surface and the distance *l* over which *α*(*E*) is positive is small, so *Q* is suppressed as per equation (3.2). It follows that the threshold field at the surface of cylinders required for breakdown *E*_*s*_ varies with the cylinder radius. The relationship between *E*_*s*_ and *R* is in the form

**Figure 2.**
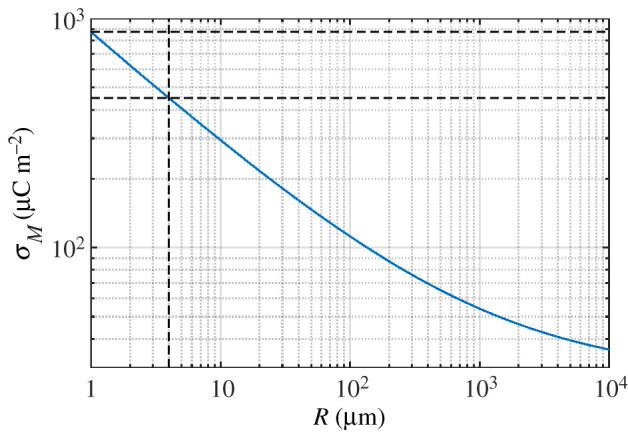
Maximum stable charge density on cylinders of radius *R* calculated using equations (3.3) and (3.4). When *R* = 1 μm, *σ*_*M*_ ≈ 873 μC m^−2^ (upper horizontal dashed line); *R* = 4 μm (vertical dashed line), *σ*_*M*_ ≈ 450 μC m^−2^ (lower horizontal dashed line). For cylinders of radius exceeding 1 cm, *σ*_*M*_ tends towards 27 μC m^−2^.

3.4 $$E_{s}(R)=A_{0}\delta \left(1+\frac{A_{1}}{\sqrt{\delta R}}\right)\;{\text{V m}}^{-1},$$

where the coefficients have been empirically determined as *A*_0_ = 31 × 10^5^ and *A*_1_ = 0.0308, and *δ* is the air density normalized to that at 298.15 K and 1 atm [22, eqn (12.16)]. From equation (3.3) with *x* = *R* and *E*_*c*_ = *E*_*s*_, the maximum surface charge density accumulated before breakdown *σ*_*M*_ = *E*_*s*_ɛɛ_0_ can be calculated for any *R* (figure 2).

## 4. Oscillating air motion force

Considering a cylinder immersed in fluid of density *ρ* and kinematic viscosity *ν* oscillating at velocity *V* and frequency *f* = *ω*/2*π*, the force on the cylinder by the fluid motion can be successfully modelled by the combination of viscous drag and fluid added mass forces:

4.1 $$F_{a}=4\pi \rho \nu G\left(V_{x}-y\frac{\text{d}\theta }{\text{d}t}\right)+\left(\pi \rho R^{2}-\frac{\pi \rho \nu G}{2gf}\right)\left(\frac{\text{d}V_{x}}{\text{d}t}-y\frac{{\text{d}}^{2}\theta }{\text{d}t^{2}}\right),$$

where

$$G=\frac{-g}{g^{2}+{\left(\pi /4\right)}^{2}},$$

g=γ+lns,

4.2 $$s=\frac{R}{2}\sqrt{\frac{\omega }{\nu }}$$

and *γ* is Euler’s constant [14,25]. The movement of the cylinder is represented by the angular displacement *θ* and velocity d*θ*/d*t*. This solution is valid provided *s* ≪ 1. Here, this constraint is met when *f* < 1 kHz, considering the bumblebee hair mechano-sensor of microscale diameter in air at 298.15 K and 1 atm (*ν* ≈ 16 × 10^−6^ m^2^ s^−1^), giving *s* < 0.04 using equation (4.2).

### 4.1. Air flow around the body

The flow velocity of fluids moving over a surface is not homogeneous. Near the surface of an object, a boundary layer forms in which fluid velocity is smaller than that of the free flow. This effect has been shown to be pertinent for insect hair operating as flow sensors—hairs that are too short to protrude out of the boundary layer are less sensitive to the air flow around the insect [19]. This effect is sensitive to the frequency of oscillation of the surrounding air medium whereby higher frequencies exhibit thinner boundary layers [14,25]. Consequently, hairs of different lengths will experience different forcing regimes due to their interactions with the non-uniform fluid boundary layer.

The boundary layer effect is considered here by finite-element modelling of the air flow around a bee body approximated to a prolate spheroid with smooth surface. Although a geometrical simplification, this should allow a first order estimation of the air flow. Air is modelled to oscillate with free-flow velocity along the *x* axis:

$$V_{0}=|V_{0}|\sin\omega t.$$

**Figure 3.**
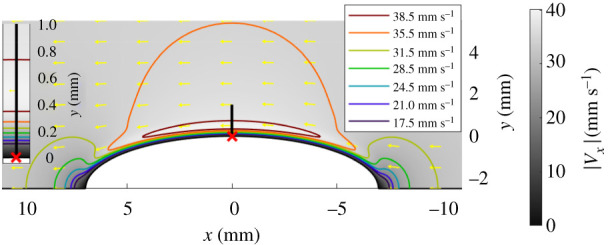
Cross-section of fluid flow over a prolate spheroid of approximate bumblebee dimensions obtained by finite-element modelling. The flow shown is driven by an oscillating free field air flow *V*_0_ in the *x* direction, |*V*_0_| = 35 mm s^−1^, *f* = 130 Hz. The greyscale colour gradient shows flow speed in the *x* direction |*V*_*x*_| at a snap-shot in time. Yellow arrows depict the direction of flow and coloured isolines show zones of equal fluid velocities. The red cross is the position of the base of the hair and the black line shows a 1.5 mm hair extending perpendicular to the body surface. (Inset) Close-up view of the boundary layer demonstrating the low flow velocity near the surface (1 mm depth) which increases with distance *y* from the surface.

Figure 3 shows the axisymmetrical cut plane with the same coordinate system as figure 1. The model treats the air as incompressible, an assumption that is also made by previous studies on fluid flow interactions with sensing hairs [19,25].

Considering a hair protruding normally from the model bee surface at the mid-point along the semi-major axis (figure 3). The torque generated by this air flow on the hair, *T*_*a*_, can then be computed using equations (4.1) and (2.1) by substituting the flow velocity, *V*_*x*_(*y*), from the finite-element modelling.

The software package COMSOL multiphysics 5.4 was used to perform the finite-element modelling. The flow field is modelled in a cylindrical ‘wind tunnel’ of 10 cm radius and 20 cm length with *V*_0_ defined as an input flow from one end of the cylinder. The model bumblebee is centred in the cylinder with semi-major axis parallel to the cylinder axis. The hair is not present in the model.

## 5. Angular displacement response of the hair

The neural transducer in the base of the hair is sensitive to the angular displacement or velocity due to the torque applied. Then, the applied torque has to be related to angular displacement to understand hair sensitivity. It is shown in this section that the angular displacement is proportionally related to the torque applied so that

1. the hair’s relative response to electic field and air motion can be found via the coupled torques and
2. the results can be generalized regardless of the mass–spring–damper properties of the machano-sensory hair.

The relationship between force applied and displacement response of a mass–spring–damper system is described by a second order ordinary differential equation [14,26]:

5.1 $$I\frac{{\text{d}}^{2}\theta }{\text{d}t^{2}}+C\frac{\text{d}\theta }{\text{d}t}+K\theta =T,$$

where *θ* is the response, *T* is the forcing function and *I*, *C*, and *K* are constants relating to the mass inertia, damping and restoring coefficients of the system respectively. For the hair pendulum, *θ* is the angular displacement and *T* is the torque applied. When *T* is a harmonic function, the steady-state solution to the response is [26]

θ(t)=|T|F(ω,I,C,K)sin(ωt+Φ(ω,I,C,K)),

where F and Φ are constants dependent on forcing frequency *ω* and the properties *I*, *C* and *K*. The overall response comprises of the natural response of the system in addition to the steady-state response. However, the natural response is independent of the forcing term [26] so is not necessary for the discussion here.

Consider a hair mass–spring–damper system that has angular displacement response *θ*_1_ when excited by harmonic function *T*_1_. When it is excited by *T*_2_ oscillating at the same frequency as *T*_1_,

5.2 $$\frac{\left|\theta_{1}\right|}{\left|\theta_{2}\right|}=\frac{\left|T_{1}\right|}{\left|T_{2}\right|}.$$

Equation (5.2) demonstrates the proportional relation between the magnitudes of hair angular displacement |*θ*| and applied torque |*T*| with the proviso that the mass–spring–damper characteristics of the system are the same. This is discussed in the following §5.1.

### 5.1. Hair and fluid motion

The relationship in equation (5.2) is valid when the hair mass–spring–damper system characteristics (*I*, *C* and *K*) are the same for both stimuli. Hair movement in a fluid experiences added mass and damping effects from the fluid which is included in equation (4.1). Rearranging equation (4.1), *F*_*a*_ can be described as having 2 components, one due to fluid motion *F*_*V*_ and another due to hair movement in the fluid $F_{\theta }$:

$$F_{a}=F_{V}(y)-F_{\theta },$$

where the components

$$F_{V}(y)=4\pi \rho \nu GV_{x}(y)+\left(\pi \rho r^{2}-\frac{\pi \rho \nu G}{2gf}\right)\frac{\text{d}V_{x}(y)}{\text{d}t}$$

and

$$F_{\theta }=4\pi \rho \nu Gy\frac{\text{d}\theta }{\text{d}t}+\left(\pi \rho r^{2}-\frac{\pi \rho \nu G}{2gf}\right)y\frac{{\text{d}}^{2}\theta }{\text{d}t^{2}}.$$

Then, equation (5.1) for oscillating air motion, using equation (2.1), is

5.3 $$\begin{array}{l} I_{h}\frac{{\text{d}}^{2}\theta }{\text{d}t^{2}}+C_{h}\frac{\text{d}\theta }{\text{d}t}+K_{h}\theta =\int_{0}^{L}F_{V}y\text{d}y-\int_{0}^{L}F_{\theta }y\text{d}y\\ \;=T_{V}-C_{\theta }\frac{\text{d}\theta }{\text{d}t}-I_{\theta }\frac{{\text{d}}^{2}\theta }{\text{d}t^{2}}, \end{array}$$

where *I*_*h*_, *C*_*h*_ and *K*_*h*_ are the hair’s mass inertia, damping and restoring coefficients and the constants

$$C_{\theta }=\frac{4}{3}\pi \rho \nu GL^{3}$$

and

$$I_{\theta }=\frac{1}{3}\left(\pi \rho R^{2}-\frac{\pi \rho \nu G}{2gf}\right)L^{3}.$$

Rearranging equation (5.3), the mass–spring–damper properties for hair moving in an oscillating air flow can be derived as $I=I_{h}+I_{\theta }$, $C=C_{h}+C_{\theta }$ and *K* = *K*_*h*_ which have contributions from both the hair mechanical characteristics and its movement in the fluid.

Recognizing that the hair is surrounded by air regardless of stimulus, the force component $F_{\theta }$ due to its movement in air should also be present for hair movement due to electric field stimulus and the mass–spring–damper properties for hair moving due to oscillating electric field are the same as those for oscillating air motion stimulus at the same frequency. A corollary of this is that the relationship in equation (5.2) is valid, allowing a comparison of the hair mechano-sensory response due to electric field and air motion stimuli by considering only the applied torques *T*_*V*_ and *T*_*E*_.

### 5.2. Relative significance of air motion and electric stimuli

The angular displacement response of bumblebee hair to air motion or electric stimuli cannot be calculated as the hair’s properties *I*_*h*_, *C*_*h*_ and *K*_*h*_ are unknown. However, their relative responses can be found from the applied torques *T*_*V*_ and *T*_*E*_ as demonstrated by the analysis above. Hence the sensitivity of the hair mechano-sensor to air motion and electric stimuli can be compared. Assuming that oscillating air disturbances produced by wing beats of nearby flying insects is a perceivable signal for the bumblebee mechano-sensory hair, the equivalent electric field stimulus can be found. Here, it is worth noting that stimuli of smaller amplitude may still be perceived and be of biological significance [27,28].

## 6. Modelling results

The flow of a sinusoidally oscillating far-field air motion over the bee body model is computed using finite-element modelling over realistic parameter ranges:

— L=0−1.5 mm to be representative of typical bumblebee hair lengths (figure 1*a*–*c*);
— bumblebee body modelled by spheroid (§4.1) with semi-major axis length *b*_maj_ = 6–8 mm and semi-minor axis length $b_{\min}=2-4\;\text{mm}$ to be representative of bumblebee body dimensions;
— $|V_{0}|=15-55\;{\text{mm s}}^{-1}$ and f=80−230 Hz to be representative of air disturbance from nearby insect wing beats [16].

**Figure 4.**
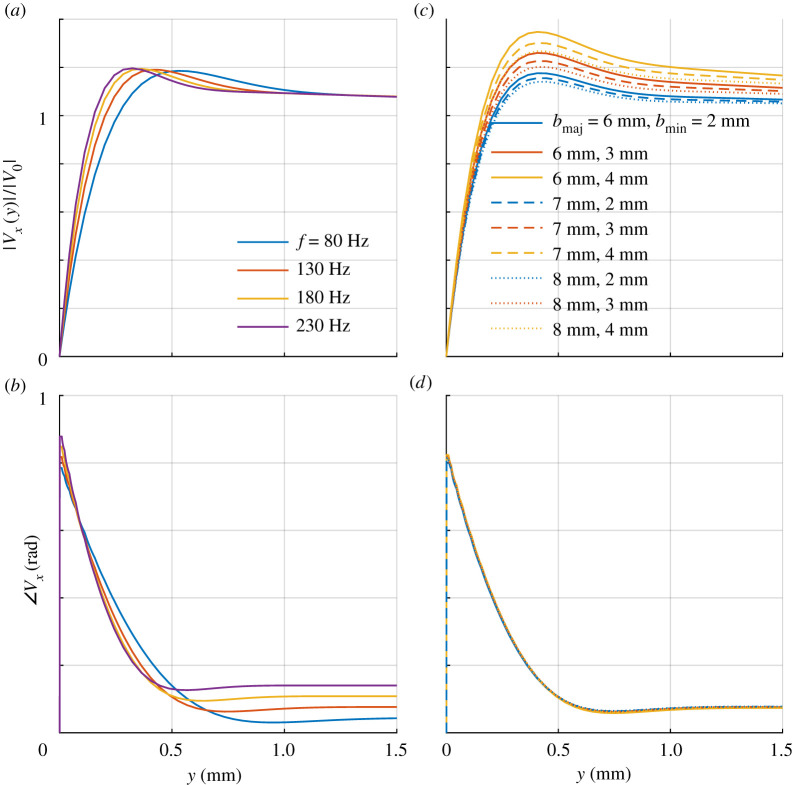
Normalized amplitude |*V*_*x*_|/|*V*_0_| and phase $∠V_{x}$ of air flow along *x* against distance *y* from bee body surface. (*a*,*b*) For a fixed body geometry, ellipsoid with *b*_maj_ = 7 mm and *b*_min_ = 2.5 mm, the boundary layer flow is seen to vary with *f*. (*c*,*d*) Boundary layer flow variation with ellipsoid dimensions *b*_maj_ and *b*_min_ at *f* = 130 Hz.

This modelling demonstrates the extent and shape of the boundary layer, depicting the small flow amplitudes near the surface, gradually increasing until reaching a maximum before decreasing towards |*V*_0_| (figures 3 and 4). Note that the boundary layer depth is 0.3–0.5 mm, commensurate with the length of many hairs on the bee’s cuticular surface.

At the position of the hair, air velocity along the *x* axis, *V*_*x*_, is found to exhibit the following characteristics:

— |*V*_*x*_(*y*)| ∝ |*V*_0_| so that the normalized amplitude |*V*_*x*_(*y*)|/|*V*_0_| at distance *y* from the surface is the same for all values of |*V*_0_|;
— boundary layer depth decreases with *f* but maximum normalized air motion velocity, |*V*_*x*_(*y*)|/|*V*_0_|, increases with *f* (figure 4*a*);
— maximum |*V*_*x*_(*y*)|/|*V*_0_| decreases with *b*_maj_ but increases with *b*_min_ (figure 4*c*).

Expected from fluid dynamic theory, these results highlight the importance of frequency and body shape in the depth and geometry of the air motion boundary layer in which sensory hairs reside.

**Figure 5.**
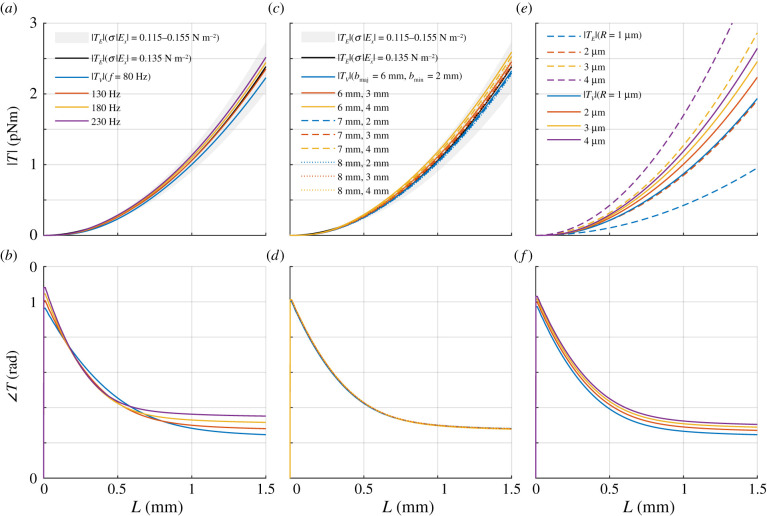
Torque amplitude |*T*| and phase ∠T variation with hair length *L*. Dependence on (*a*,*b*) frequency *f*, (*c*,*d*) body dimensions *b*_maj_ and *b*_min_ and (*e*,*f*) hair radius *R*. Results calculated with |*V*_0_| = 35 mm s^−1^, *f* = 130 Hz, *b*_maj_ = 7 mm, *b*_min_ = 2.5 mm and *R* = 2.5 μm unless specified. |*T*_*V*_| variation with *f*, *b*_maj_ and *b*_min_ falls within the variation of |*T*_*E*_| with *σ*|*E*_*x*_| between 0.115 and 0.155 N m^−2^, corresponding to $|E_{x}|=400-500\;{\text{V m}}^{-1}$ if *σ* = 270 μC m^−2^. |*T*_*E*_| dependency on *R* is much larger than |*T*_*V*_|.

The corresponding *T*_*V*_ generated by the boundary layer flow reflects the characteristics of *V*_*x*_ (figure 5). Further, |*T*_*V*_| shows stronger dependency on *L* or *R* than *f*, *b*_maj_ or *b*_min_ for the parameter ranges considered.

According to equation (3.1), |*T*_*E*_| is invariant to *f*, *b*_maj_ or *b*_min_ but is dependent on *L* and *R*. Additionally, |*T*_*E*_| varies with *σ*|*E*_*x*_|. From equation (5.2), the equivalent hair angular displacement response due to an electric field can be found by equating *T*_*V*_ = *T*_*E*_ and using equation (3.1), the corresponding *σ*|*E*_*x*_| can be calculated. Variation in |*T*_*E*_| for $\sigma |E_{x}|=0.115-0.155\;{\text{N m}}^{-2}$ is shown as the shaded areas in figure 5*a*,*c*. These values of |*T*_*E*_| are comparable to |*T*_*V*_| for the same hair dimensions, *R* = 2.5 μm and L=0−1.5 mm, and over the range of *f* and body dimensions *b*_maj_ and *b*_min_ considered. Then, for |*T*_*E*_| to be comparable to |*T*_*V*_|, the electric field stimulus |*E*_*x*_| must be $4000-6000\;{\text{V m}}^{-1}$ if *σ* ≈ 27 μC m^−2^, but if *σ* ≈ 270 μC m^−2^ (<*σ*_*M*_), then |*E*_*x*_| only needs to be $400-500\;{\text{V m}}^{-1}$.

Further, both stimuli exhibit similar dependence on *L* (figure 5*a*,*c*). Notably, sensitivity of |*T*_*E*_| to *R* is larger than |*T*_*V*_| (figure 5*e*), which can be attributed to the variation in charging surface area being larger than that in drag coefficient for the same change in *R*.

## 7. Discussion

The analysis presented here establishes that an electric field can apply a torque on a filiform hair that is comparable to that of the air motion from insect wing beats. The oscillating air motion modelled here is analogous to air disturbances produced by the wing beats of insects flying nearby, at 0.01 to 1 m distance depending on the insect species and orientation. While present around any flying insect, such air motion is characteristic of the buzzing sound surrounding flying bees. These acoustic emissions are generally considered to be detectable by filiform hair sensors and therefore perceptible by bees, and it is proposed here that an oscillating electric stimulus producing comparable torque on the hair would be similarly perceived.

### 7.1. Frequency response

One significant difference between air motion and electric stimuli is the frequency dependence of the applied torque. While boundary layer flow effects mean *T*_*V*_ is frequency dependent, *T*_*E*_ has a flat frequency response (figure 5*a*,*b*). More specifically, the frequency response of the transfer function between the applied electric field *E*_*x*_ and the electric torque *T*_*E*_ is flat, but between *V*_0_ and *T*_*V*_ is not. Note, however, that the transfer function between the forcing torque *T* and the angular displacement response *θ* is frequency dependent due to the inherent mass–spring–damper properties of the hinge and its movement against the surrounding air (§5). Therefore, when applying an electric field stimulus *E*_*x*_, one would observe a frequency response of the angular displacement due only to the mass–spring–damper properties of the hinge and the hair’s movement. This straightforward electric field to hair displacement coupling has been used in previous experiments to investigate mechanical transduction in dipteran acoustic receivers [29]. In contrast, when applying an air motion stimulus *V*_0_, the frequency response of the air flow over the body is added to the frequency response from the mechanics of the hair. This has been pointed out in previous observations [18].

This difference between air motion and electric stimuli coupling to the hair means that the hair mechano-sensor may select for one stimulus mode over the other depending on the frequency range of the signal. For example, at low frequencies or slow variations in air flow velocity, the boundary layer depth is large so |*T*_*V*_| generated by |*V*_0_| is smaller than at higher frequencies, but because the electric stimulus is invariant in frequency, the corresponding |*T*_*E*_| can be generated by a smaller |*E*_*x*_| than at higher frequencies. Conversely, since |*T*_*V*_| is larger for increasing frequencies (figure 5*a*), the equivalent electric field stimulus amplitude |*E*_*x*_| must also be larger with increasing frequency. However, a physical phenomenon not considered in the modelling here is that the displacement of the air particles oscillating at |*V*_0_| decreases with increasing frequency [30]. Since hair displacement is limited by the displacement of the air particles, the coupling between air motion and hair displacement will reduce above some frequency regardless of |*T*_*V*_|, a limitation that does not affect electric stimulus. Then, it is possible that bumblebee hair may be designed to be a bi-modal sensor, selectively responding to air motion or electric field at different frequencies.

### 7.2. Substrate and hair morphology

The results show variation of |*T*_*V*_| on body dimensions (figure 5*c*) while, from equation (3.1), there is no effect on |*T*_*E*_|. Dependence of air flow on the shape of the body cross-section was shown previously using the comparison between air flow across a cylinder and along a cylinder [19]. Then, air disturbance moving along the semi-minor axis would encounter a different body cross-section from when moving along the semi-major axis (as modelled here). The resulting difference in boundary layer flow and hence |*T*_*V*_| produces a directionality to the hair response that is absent for electric stimuli.

Admittedly, the overall shape of a bee is not a simple ellipsoid. Deviations from the idealized shape considered here are expected to affect the flow modelling. The present approach also considers the mechanics of an isolated hair which would be different from a hair situated within a population of hairs that sometimes takes the form of a dense fur-like cover, such as that of bumblebees (for example, figure 1*c*). Viscous air coupling between neighbouring hairs in a dense array tends to reduce the angular displacement response of the hairs to air flow with the effect being more pronounced at lower frequencies [31–34]. Modelling the response of a hair in a fur-like scenario would introduce a level of complexity beyond the scope of this study.

Variation in hair geometry is likely to facilitate one sensing mode over the other. The modelling shows that hairs with larger radius have a larger response to electric field stimulus due to the increase in surface area for charging (figure 5*e*). For a cylindrical geometry, the corresponding increase in |*T*_*V*_| is smaller for the same change in *R*. Bee hairs exhibit morphological features at the microscale, such as curvature, tapering and branching. While the detailed features and their variability can be observed, they are difficult to quantify. Yet, these features affect the surface area for air motion to act on and for charges to accumulate. For example, fluid flow forces experienced by sensilla on the antennule of the freshwater crayfish are modulated by their morphology so that feathered sensilla can be subject to fluid flow forces a magnitude larger than tapered sensilla [35]. Similarly, morphology affects the surface area available for electric charges to accumulate and hence the electric force coupled to the hair since *F*_*E*_ = *qE*. So, branching increases both electric and air motion coupling to the hair. It is possible that a branched geometry can change the relative magnitude of the response to electric field against air motion. Further, it can be speculated that an array of verticillate hairs could serve to promote the trapping of air inside their canopy while enhancing the electric field sensing mode by increasing charge accumulation.

### 7.3. Electric charging of the hair

If hair surface charge density can reach the air breakdown limit *σ*_*M*_ > 450 μC m^−2^ (§3.1), then the |*E*_*x*_| required to produce a comparable response to insect wing beats is approximately 300 V m^−1^. However, even though *σ*_*M*_ is high, stable charge densities on the hair would depend on the leakage rate balanced against the charging rate. Charging mechanisms and rates for bee hairs in natural scenarios are as yet unknown. In addition, there is considerable uncertainty regarding the charge density distribution on the hair surface. The charge distribution is dependent on unknown factors such as the charging mechanism and hair surface conductivity. It is also likely that individual hair charge varies widely—supported by empirical observations of variability in charge levels (up to 3 orders of magnitude) on whole bees [1–3].

An important additional factor is that a bee is likely not to be homogeneously charged. Even on a small surface such as the hair, the charge distribution is likely not to be uniform. Realistic values are difficult to estimate as measurements of charge density over such fine spatial resolution and geometrical complexity are yet to be made. Bumblebees have been estimated by several independent studies to carry a charge of approximately 30 pC. This figure suggests that the average amount of charge per hair cannot be large. Although negative charge regions may exist on the bee surface to offset the net bee charge, this would mean very strong electric fields forming due to the close proximity and charge magnitudes. It can be suggested here that only some hairs are liable to accumulate charge densities that enables their operation as electric field sensors.

### 7.4. Possible electric signals

It is convenient in theoretical analysis to consider harmonic forcing, but signals in realistic scenarios often deviate considerably from pure tone harmonics. An arbitrary free field flow *V*_0_ signal can be specified in the finite-element modelling to calculate the boundary layer flow *V*_*x*_ and hence the air motion forcing term via equations (4.1) and (2.1). The function for the air motion forcing can then be compared to the electric forcing term in equation (3.1) so the equivalent electric field stimulus *E*_*x*_ can be found.

Taking the |*E*_*x*_| required to produce perceivable stimulus as 300 V m^−1^, it is possible to speculate on the information available via this sensing mode. The natural electric field in the atmosphere near ground is typically approximately 120 V m^−1^ with little variability (hourly standard deviation approx. 10 V m^−1^) in fair weather [36]. However, in unstable weather conditions this can increase to approximately 1000 V m^−1^, representing an electric field variation that can produce torque on the hair, |*T*_*E*_|, larger than that produced by air disturbances of insect wing beats. Consequently, the possibility exists that useful information can be conveyed in the electric environment.

It is noteworthy that the electric stimulus acting on the bee hair can also come from the charge on the hair itself. A charge in space would induce equal and opposite image charges on any grounded object. Similarly, an approaching charged bee would induce image charges on a flower. At a close distance of several body lengths, it is likely that the electric field from the image charges is perceptible by the bee hair. This scenario highlights the relevance of electrostatic effects taking place as a bee, or any insect carrying a charge, approaches a flower or any grounded or electrically polarizable object. Also, the electric stimulus is poised to rapidly increase as distance gets shorter between bee and flower, a rate of increase that may contain information for the bee’s flight control and foraging strategy. It was previously shown that bumblebees can detect and learn different structures of floral electric fields [3], and as such the geometry of the image charge, and its modifications could help bees identify floral morphology, or deviations thereof.

## 8. Summary and conclusions

A physical mechanism for electric actuation of bumblebee hair is described based on previously reported observations. From this description, response characteristics of isolated hair to electric fields are derived and compared to that due to oscillating air motion. It is pointed out that when comparing hair response between oscillating electric field and air motion stimuli, it is sufficient to compare only the torque applied by the stimulus regardless of the hair’s mass–spring–damper characteristics or hair motion in still air, meaning that the analysis can be generalized to all stiff hairs as long as the small angle motion condition is met.

An important point of difference between electric field and air motion stimuli is in the frequency response of the torque applied. There is an increase with frequency of the torque amplitude applied by air motion because of boundary layer effects while the torque amplitude applied by electric field is constant with frequency. Previous observations of differences in hair frequency response measured with the different physical stimuli [18] can be attributed to the described mechanism and highlights the need to make a distinction between frequency responses for different sensing modes.

Additionally, the response amplitude to air motion is influenced by the geometry of the body the air is flowing over. This factor introduces geometrical considerations that are not trivial to model. One important geometrical arrangement is the position of a hair in an array, a feature common in bumblebees and other pollinators. It is suggested that this arrangement reduces air motion sensitivity, particularly at low frequencies [31–34], thus making the electric field sensing modality more prevalent.

An increase in radius for the cylindrical hair model increases the charging surface area more than the surface area on which air motion acts. This results in an increase in the electric field response that is larger than the air motion response for the same change in hair radius, favouring the electric sensing modality. It follows that morphological features of bee hairs deviating from the cylindrical geometry, for example branching, may produce the same effect.

Air disturbances from nearby insect wing beats are taken here as a relevant signal that is perceived by bumblebees. This signal is approximated here as oscillating air flows $15-55\;{\text{mm s}}^{-1}$ at 80–230 Hz [16]. Taking the typical air flow signal to be 35 mm s^−1^ at 130 Hz, a similar hair movement response amplitude can be elicited by an applied oscillating electric field of greater than 300 V m^−1^ depending on the charge density distribution on the hair. The minimum oscillating electric field strength of approximately 300 V m^−1^ is based on the air breakdown limit for cylindrical hair of micrometre scale radius 4 μm enabling large surface charge densities (approx. 450 μC m^−2^) to accumulate. In practice, there is considerable uncertainty in the surface charge on the hair and its density distribution which are crucial in establishing the sensitivity of the hair electric field sensor.

This theoretical investigation here highlights the possibility of bi-modal sensing of both oscillating electric field and air motion via the bumblebee hair mechano-sensor. The features of and differences between both sensing modes should be considered in future investigations of the ecological relevance and functions of bumblebee hair electric field or air motion sensing.

## Data accessibility

No specific algorithms or datasets were used in this study.
